# Rhomboid domain containing 1 promotes colorectal cancer growth through activation of the EGFR signalling pathway

**DOI:** 10.1038/ncomms9022

**Published:** 2015-08-24

**Authors:** Wei Song, Wenjie Liu, Hong Zhao, Shangze Li, Xin Guan, Jianming Ying, Yefan Zhang, Fei Miao, Mengmeng Zhang, Xiaoxia Ren, Xiaolu Li, Fan Wu, Yuechao Zhao, Yuanyuan Tian, Wenming Wu, Jun Fu, Junbo Liang, Wei Wu, Changzheng Liu, Jia Yu, Shudong Zong, Shiying Miao, Xiaodong Zhang, Linfang Wang

**Affiliations:** 1 Department of Biochemistry and Molecular Biology, State Key Laboratory of Medical Molecular Biology, Institute of Basic Medical Sciences Chinese Academy of Medical Sciences, Peking Union Medical College, Beijing 100005, China; 2 Department of Abdominal Surgical Oncology, Cancer Hospital & Institute, Chinese Academy of Medical Sciences, Beijing 100021, China; 3 College of Life Sciences, Wuhan University, Wuhan 430072, China; 4 Department of Pathology, Cancer Hospital & Institute, Chinese Academy of Medical Sciences, Beijing 100021, China; 5 Department of General Surgery, Peking Union Medical College Hospital, Chinese Academy of Medical Sciences, Beijing 100730, China; 6 National Research Institute for Family Planning, WHO Collaboration Center of Human Reproduction, Beijing 100081, China

## Abstract

Rhomboid proteins perform a wide range of important functions in a variety of organisms. Recent studies have revealed that rhomboid proteins are involved in human cancer progression; however, the underlying molecular mechanism remains largely unclear. Here we show that RHBDD1, a rhomboid intramembrane serine protease, is highly expressed and closely associated with survival in patients with colorectal cancer. We observe that inactivation of RHBDD1 decreases tumor cell growth. Further studies show that RHBDD1 interacts with proTGFα and induces the ADAM-independent cleavage and secretion of proTGFα. The secreted TGFα further triggers the activation of the EGFR/Raf/MEK/ERK signalling pathway. Finally, the positive correlation of RHBDD1 expression with the EGFR/Raf/MEK/ERK signalling pathway is further corroborated in a murine model of colitis-associated colorectal cancer. These findings provide evidence of a growth-promoting role for RHBDD1 in colorectal cancer and may aid the development of tumor biomarkers or antitumor therapeutics.

## Abstract (web summary)

Rhomboid proteins are involved in human cancer progression. Here, the authors show that RHBDD1, a rhomboid intramembrane serine protease, promotes tumor growth in colorectal cancer via cleavage and secretion of TGFα, and activation of the EGFR/Raf/MEK/ERK signalling pathway.

## Graphical abstract

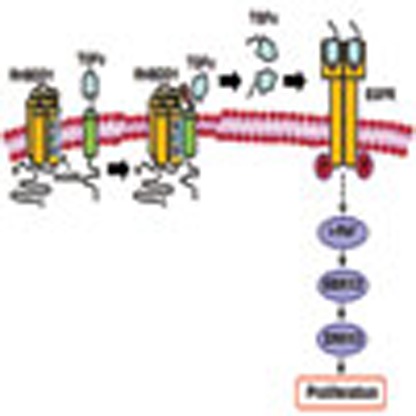

Colorectal cancer (CRC) is the third most common cancer in men and the second in women worldwide, and it is estimated to be the fourth leading cause of cancer-related death^1^. In recent years, there have been many encouraging research achievements in this field; however, the molecular mechanism of tumorigenesis and local tumor growth in the colon remains largely unclear.

Transforming growth factor α (TGFα), a member of the epidermal growth factor (EGF) family, is a type I transmembrane protein that is expressed on the cell surface and can be cleaved and activated by transmembrane proteases^2^. Previous evidence indicates that the activation of TGFα is mediated by TNF-Alpha Converting Enzyme (TACE) in mammals^3^. The active form of TGFα binds to its only receptor, EGF receptor (EGFR), to activate the EGFR signalling pathway^4^. Studies have shown that TGFα is an important determinant of abnormal growth in malignant progression. Transgenic mice overexpressing TGFα show hyperplasia and malignancy in some tissues^5^,^6^, and the ectopic expression of TGFα causes the malignant progression of human tumours in nude mice^7^. Although it plays a significant role in tumor progression, the mechanism by which TGFα confers a growth advantage to tumor cells and that by which TGFα is activated in tumor cells are still not completely understood.

Rhomboid family proteins are intramembrane serine proteases that are evolutionarily conserved from bacteria to humans^8^. Rhomboid-1, the best characterized member of the family, cleaves the TGFα-like ligand Spitz to regulate EGFR signalling in *Drosophila*^9^,^10^. In *Caenorhabditis elegans*, the rhomboid homologue ROM-1 is also implicated in the proteolytic release of the EGF-like ligand Lin-3 (ref. 11). However, in mammals, the majority of the existing data show that the cleavage and release of EGF family ligands are primarily achieved by ADAM family metalloproteases^3^,^12^. An important question addressed here is whether the essential role of rhomboid in *Drosophila* is recapitulated in mammals. In contrast to earlier reports, rhomboid-like 2 (RHBDL2) has recently been found to cleave EGF, facilitate its secretion and trigger the activation of EGFR^13^. This is the first evidence that mammalian rhomboid proteases are directly involved in EGFR signalling. In addition, EGFR hyperactivity is implicated in many cancers^14^, and rhomboid proteins, such as RHBDF1 and RHBDD2, are reported to be highly expressed in human cancers^15^,^16^,^17^. These studies suggest possible links between rhomboids, EGFR signalling and cancer in mammals.

In this study, we seek to investigate the oncogenic role of rhomboid family member RHBDD1 in CRC. We hypothesize that RHBDD1 may be important for EGFR pathway activation, and accordingly, the growth of CRC cells. And more importantly, our results support a role for RHBDD1 as a novel prognostic marker or therapeutic target in human CRC.

## Results

### RHBDD1 upregulation is associated with poor prognosis in CRC

**Figure 1:**
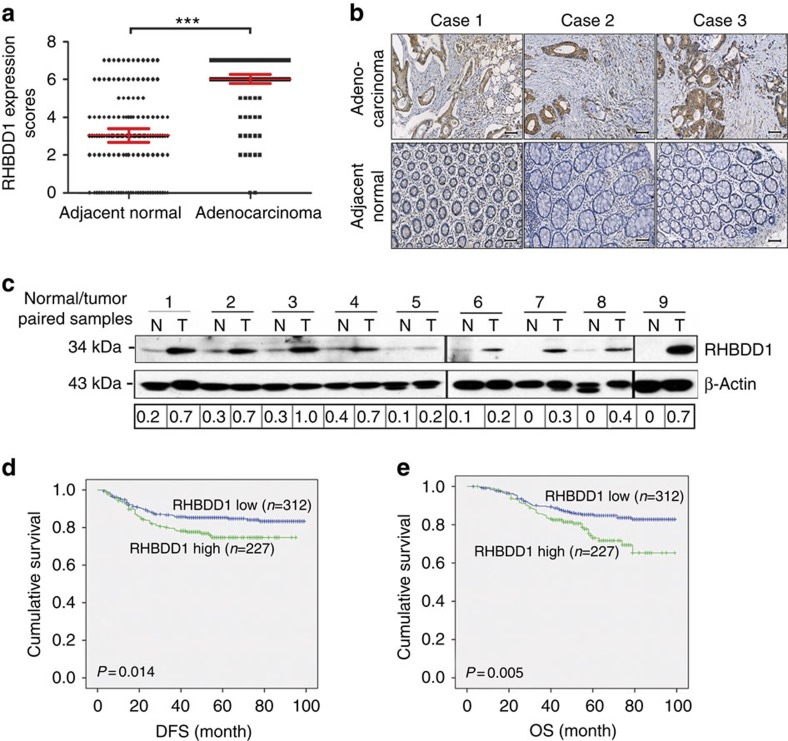
RHBDD1 expression in specimens from CRC patients. (**a**) Tissue microarray data analysis of RHBDD1 expression in 142 CRC patients. The data are presented as means±s.d., *N*=142, the Wilcoxon signed rank test, ***P<0.001. (**b**) Representative immunohistochemical staining of RHBDD1 on tissue microarrays containing CRC tissues and adjacent normal tissues (scale bar, 50 μm). (**c**) Expression of RHBDD1 in CRC patients was analysed by western blotting using β-actin as a loading control. (**d**) and (**e**) Kaplan–Meier survival analysis of the correlation between RHBDD1 expression and disease-free and overall survival in 539 CRC patients (*N*=539, the Kaplan–Meier method with log-rank testing, DFS: *P*=0.014; OS: *P*=0.005).

To investigate the oncogenic role of RHBDD1 in CRC progression, we first produced an in-house anti-RHBDD1 mouse monoclonal antibody with high specificity (Supplementary Fig. 1a,b). Then we compared the expression levels of RHBDD1 in CRC and adjacent normal tissues using tissue microarrays containing 142 CRC samples (Supplementary Table 1). RHBDD1 expression was significantly upregulated in CRC samples compared with adjacent normal tissues (*N*=142, the Wilcoxon signed rank test, *P*<0.001, Fig. 1a). Both immunohistochemistry and immunofluorescence analysis showed that RHBDD1 was readily detectable in most CRC samples but was weakly detected in the adjacent normal tissues (Fig. 1b, Supplementary Fig. 1c). We next compared RHBDD1 expression in CRC and adjacent normal tissues of nine patients using immunoblot analysis and obtained the same results (Fig. 1c). To investigate whether the RHBDD1 expression level in tumours is associated with prognosis, we analysed the correlations between RHBDD1 expression and disease-free survival (DFS) and overall survival (OS) in 539 CRC patients who underwent resection at the Cancer Hospital, Chinese Academy of Medical Sciences, from January 2005 to December 2008. We found that patients with low RHBDD1 expression had better DFS and OS times (*N*=539, the Kaplan–Meier method with log-rank testing, DFS: *P*=0.014, OS: *P*=0.005, Fig. 1d,e). These data suggest that RHBDD1 has potential clinical value as a predictive biomarker for disease outcome in CRC.

### Correlation between RHBDD1 and clinical parameters

We next analysed the correlation between the RHBDD1 expression and the clinicopathological parameters of 539 CRC patients (Table 1). The results showed that RHBDD1 expression was closely associated with higher pathological tumor-node-metastasis (pTNM) stage (*N*=539, the *χ*^2^ test, *P*=0.0001), higher pathological node (pN) classification (*N*=539, the *χ*^2^ test, *P*=0.0002) and lower differentiation (*N*=539, the *χ*^2^ test, *P*=0.006). No significant correlation was found between RHBDD1 expression and other parameters including gender, age, history of smoking or alcohol abuse, family history of digestive system tumours, necrosis, lymphatic vessel invasion or pathological tumor (pT) staging.

### RHBDD1 inactivation decreases tumor cell growth

**Figure 2:**
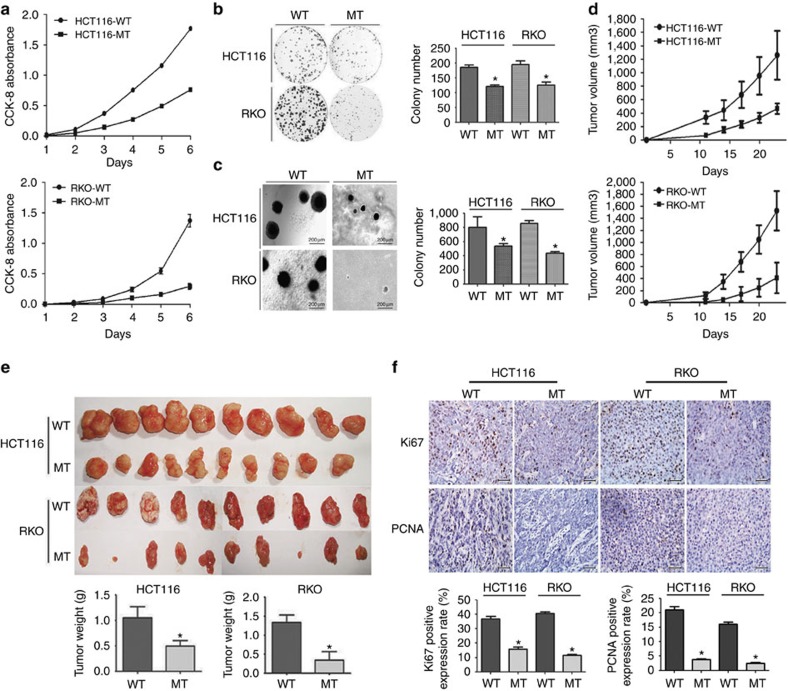
The effect of RHBDD1 inactivation on tumorigenesis *in vitro and in vivo*. (**a**) Cell proliferation assays. The samples were assayed in triplicate. Each point represents the mean value from three independent samples. (**b**) Colony formation assays and (**c**) soft agar colony formation assays. Representative photographs and bar graphs are from three independent experiments. The data are presented as means±s.d., *N*=3, the Student's two-tailed *t*-test, *P<0.05. (**d**) Growth curves of xenograft tumours. Tumor volumes were monitored every 3 days by measuring tumor diameters. The data are presented as means±s.d., *N*=10. (**e**) Images and weights of xenograft tumours. The tumours were removed, photographed and weighed. The bar graphs represent means±s.d., *N*=10, the Student's two-tailed *t*-test, *P<0.05. (**f**) Immunohistochemical analysis of xenograft tumours. Xenograft tumours were immunostained to detect Ki67 or PCNA expression (scale bar, 50 μm). The bar graphs represent semi-quantification of Ki67- and PCNA-positive cells from 4 different groups (means±s.d., *N*=6, the Student's two-tailed *t*-test, *P<0.05).

We first examined RHBDD1 expression in CRC cell lines. The result showed that RHBDD1 was upregulated in all cell lines compared with CRC adjacent normal tissue samples (Supplementary Fig. 2a). HCT116 and RKO cells inactivating endogenous RHBDD1 were then established using an improved somatic cell knock-in method (Supplementary Fig. 3a). Sequencing of genomic DNA and cDNA verified that the targeted cells were homozygous for the mutation. Both reverse transcription–PCR (RT–PCR) and real-time PCR analysis showed that the cells expressed equivalent amounts of the wild-type and mutant genes, but immunoblot analysis did not detect any trace of the RHBDD1 protein in the targeted mutant cells. It was reported that the mutation of a protein might lead to its instability and degradation through the ubiquitin/proteasome-dependent pathway^18^. Accordingly, when the mutant HCT116 and RKO cells were treated with two different proteasome inhibitors, Velcade and MG132, the RHBDD1 protein could be detected, indicating that the mutation in RHBDD1 resulted in its degradation through the proteasome pathway (Supplementary Fig. 3b). Strikingly, we found that RHBDD1 inactivation inhibited tumor cell growth in proliferation assays and colony formation assays (Fig. 2a,b). The role of RHBDD1 in reducing tumor cell growth was further validated by gene knockdown experiments (Supplementary Fig. 2b–d). We then performed soft agar assays to test whether mutation of RHBDD1 could affect anchorage-independent growth. As shown in Fig. 2c, mutation of RHBDD1 resulted in a significant decrease in colony size and quantity in both cell lines.

To further determine whether RHBDD1 affects tumorigenesis *in vivo*, we subcutaneously injected wild-type and RHBDD1-mutant HCT116 and RKO cells into nude mice and monitored tumor growth. As shown in Fig. 2d, the ability of mutant HCT116 and RKO cells to form tumours was significantly reduced compared with wild-type cells over the same period. The tumours originating from wild-type HCT116 and RKO cells reached over 1,500 mm^3^ and 1,200 mm^3^, respectively, within 23 days, whereas the tumours originating from RHBDD1-mutant cells were <600 mm^3^ at the end of the experiment. Accordingly, the weights of tumours from RHBDD1-mutant HCT116 and RKO cells decreased more than 2- and 3-fold, respectively, compared with those originating from wild-type cells (Fig. 2e). The immunohistochemical analysis showed a significant decrease in Ki67 and proliferating cell nuclear antigen (PCNA) expression in tumours derived from mutant cells compared with those derived from wild-type cells (Fig. 2f). Finally, we performed the rescue experiments to exclude off-target effects. The results from proliferation assays, clony formation assays, soft agar assays and tumorigenicity assays all showed that reintroduction of RHBDD1 recombinant protein significantly rescued the growth inhibition caused by RHBDD1 inactivation (Supplementary Fig. 4). Taken together, these *in vitro* and *in vivo* experiments demonstrate that RHBDD1 inactivation inhibits tumor cell growth and that this inhibition can largely be attributed to the RHBDD1-mediated positive regulation of cell proliferation.

### The subcellular localization of RHBDD1 in tumor cells

**Figure 3:**
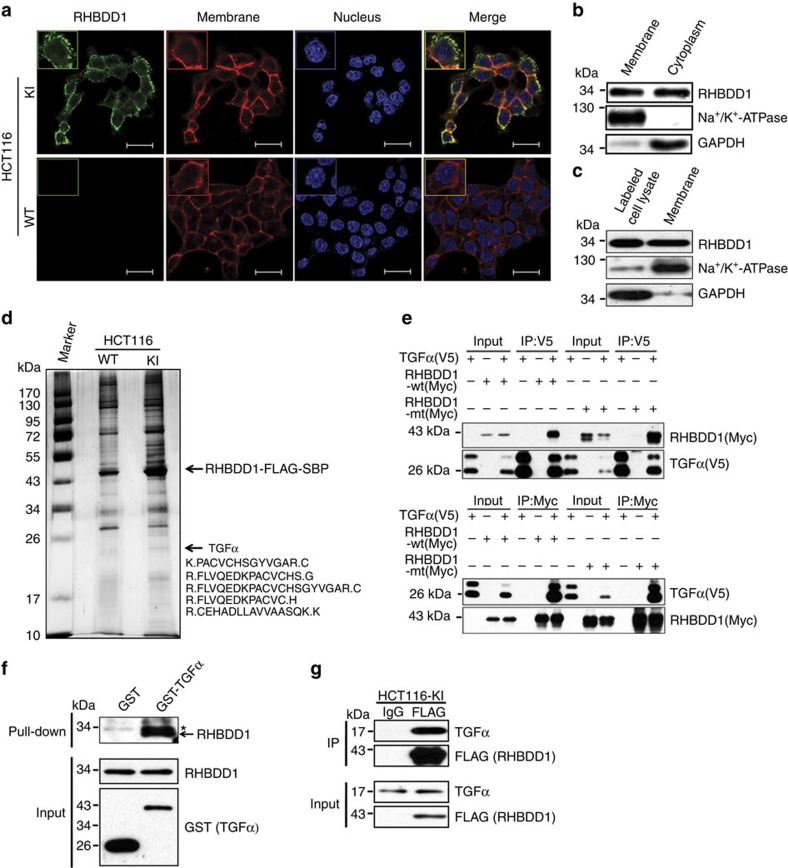
RHBDD1 is physically associated with proTGFα. (**a**) Immunofluorescence staining for RHBDD1 localization. RHBDD1 localization was analysed by immunofluorescence with anti-FLAG antibody (green). Cell membranes and nuclei were stained with Alexa Fluor 594 WGA (red) and DAPI (blue), respectively (scale bars, 22 μm). (**b**) Western blot analysis of isolated plasma membrane and cytosolic proteins in wild-type HCT116 cells to determine RHBDD1 localization. Na^+^/K^+^-ATPase and GAPDH were used as a plasma membrane marker and a cytosolic marker, respectively. (**c**) Western blot analysis of isolated cell surface proteins in wild-type HCT116 cells to determine the plasma membrane localization of RHBDD1. Na^+^/K^+^-ATPase and GAPDH were used as a plasma membrane marker and a cytosolic marker, respectively. (**d**) The affinity purification and Mass spectrometry analysis of RHBDD1-associated proteins. (**e**) Co-immunoprecipitation assays for RHBDD1 and proTGFα. proTGFα-V5 was transfected into HEK 293T cells with RHBDD1-Myc or its inactive mutant. The immune complexes (IP) and 5% input were analysed by immunoblotting with anti-Myc or anti-V5 antibody. (**f**) GST pull-down assays for RHBDD1 and proTGFα. HCT116 cell lysates were incubated with GST and GST-proTGFα fusion proteins. The GST pull-down products were analysed by immunoblotting with anti-RHBDD1 antibody. The asterisk indicates a nonspecific band. (**g**) Immunoprecipitation assays for RHBDD1 and proTGFα. Cell lysates from targeted knock-in HCT116 cells were immunoprecipitated with anti-FLAG antibody or control IgG. The immune complexes (IP) and 5% input were analysed by immunoblotting with anti-proTGFα or anti-FLAG antibody.

We next observed the subcellular localization of RHBDD1 in tumor cells. We tagged one allele of the endogenous RHBDD1 locus in HCT116 cells by knock-in of FLAG and SBP epitopes to the COOH terminus of the protein (Supplementary Fig. 3c) and then monitored the localization of RHBDD1 by immunofluorescence using an anti-FLAG antibody in HCT116 knock-in and wild-type cells. As shown in Fig. 3a, RHBDD1 was localized in both plasma membrane and cytoplasm of HCT116 cells, and it was mainly localized in the filopodial-like projections in plasma membrane but distributed equally in cytoplasm of HCT116 cells. Western blot analysis of isolated plasma membrane and cytoplasm proteins from wild-type HCT116 cells further confirmed the RHBDD1 localization (Fig. 3b). In addition, the plasma membrane localization of RHBDD1 in wild-type HCT116 cells was further confirmed by the cell surface biotinylation assay (Fig. 3c).

### RHBDD1 is physically associated with proTGFα

To further study the molecular function of RHBDD1, we employed affinity purification and mass spectrometry to identify the proteins associated with RHBDD1 in targeted knock-in HCT116 cells. Cellular extracts were prepared and subjected to affinity purification using an anti-FLAG affinity gel. The purified protein complex was resolved on SDS–polyacrylamide gel electrophoresis (PAGE) and silver stained (Fig. 3d). Mass spectrometric analysis showed that RHBDD1 was co-purified with a group of proteins, among which proTGFα was the best candidate protein due to its highest protein score calculated by BioWorks software (Supplementary Table 2). We then verified the specific interaction between RHBDD1 and proTGFα by co-immunoprecipitation assays. As shown in Fig. 3e, proTGFα-V5 interacted with both RHBDD1wt-Myc and RHBDD1mt-Myc in bidirectional co-immunoprecipitation experiments. In contrast, other EGFR ligands including EGF, HB-EGF, amphiregulin (AREG), betacellulin (BTC) and epiregulin (EPR) did not interact with RHBDD1 (Supplementary Fig. 5a–e). To further confirm that RHBDD1 binds to proTGFα, we performed a glutathione S-transferase (GST) pull-down assay. As shown in Fig. 3f, RHBDD1 physically interacted with GST-proTGFα; no binding was detected between GST and RHBDD1. Finally, the interaction between RHBDD1 and proTGFα was detected in targeted knock-in HCT116 cells. As shown in Fig. 3g, immunoprecipitation assays demonstrated that RHBDD1 was associated with endogenous proTGFα.

### RHBDD1 induces cleavage and secretion of proTGFα

**Figure 4:**
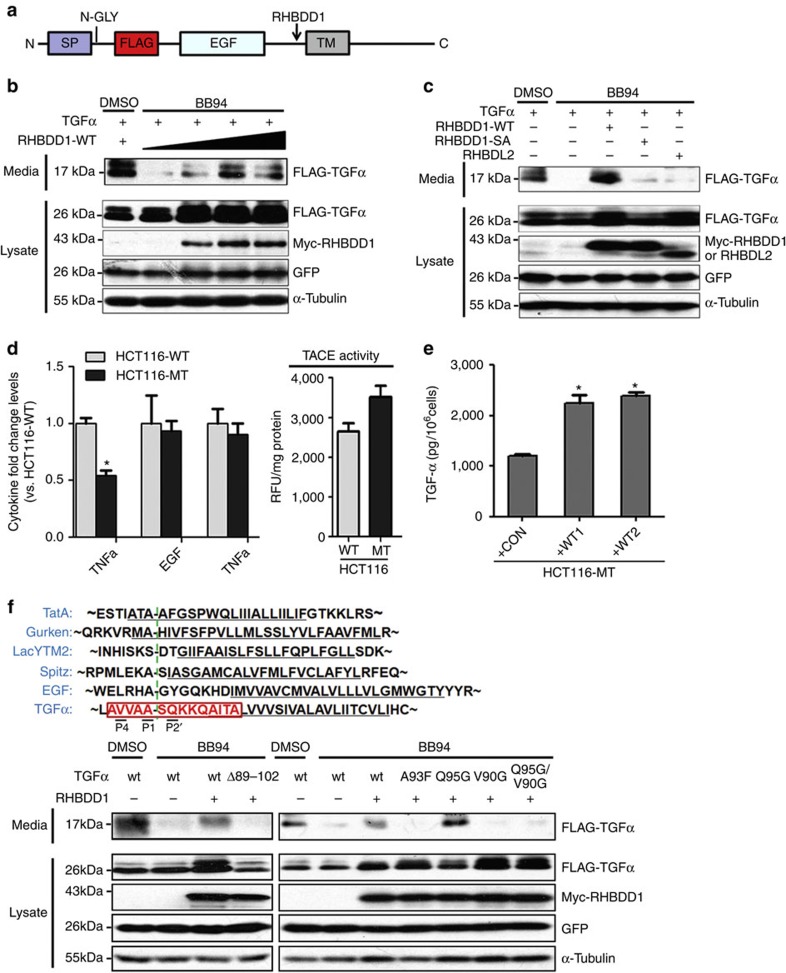
RHBDD1 induces the specific cleavage and ADAM-independent secretion of proTGFα. (**a**) Schematic of the proTGFα domain structure and the position of the FLAG tag. The RHBDD1-induced cleavage site and the N-glycosylation site were indicated. (**b**) RHBDD1 induces the ADAM-independent secretion of TGFα in a dose-dependent manner. HEK 293T cells were transfected with proTGFα-FLAG (1 μg) and RHBDD1-Myc (0, 250, 500 or 750 ng) constructs. The secretion assay was performed in serum-free medium containing 10 μM BB94. The proteins in culture supernatants were precipitated by TCA, recovered, solubilized in loading bufferand analysed by western blotting.GFP and α-tubulin were used as the transfection and loading controls, respectively. (**c**) RHBDD1 induces the ADAM-independent secretion of TGFα in an enzymatic activity-dependent manner. HEK 293T cells were transfected with proTGFα-FLAG (1 μg) and RHBDD1-WT, RHBDD1-SA or RHBDL2 (500 ng) constructs. The experiment was then performed as described in (**b**). (**d**) RHBDD1 specifically induces endogenous TGFα secretion. Media from wild-type or mutant HCT116 cells were collected and assayed for soluble TGFα, EGF and TNFα levels by ELISA, and the relative fold changes were plotted. The data are presented as means±s.d., *N*=3, the Student's two-tailed *t*-test, *P<0.05. (**e**) Media from mutant HCT116 cells in which RHBDD1 recombinant protein was re-introduced were collected, and soluble TGFα level was measured by ELISA. The data are presented as picograms per 10^6^ cells±s.d. of three independent experiments. The data are presented as means±s.d., *N*=3, the Student's two-tailed *t*-test, *P<0.05. (**f**) RHBDD1 cleaves proTGFα within the juxtamembrane region at Ala 93. An alignment of the rhomboid recognition motifs in TatA, Gurken, Lac YTM2, spitz and EGF with the corresponding residues of proTGFα. The cleavage region is highlighted and boxed in red, and the cleavage site is indicated in a green dotted line. HEK 293T cells were transfected with Myc-tagged RHBDD1 (500 ng) and FLAG-tagged proTGFα, proTGFα deletion (Δ89–102) or proTGFα mutation (A93F, Q95G, V90G, Q95G/ V90G and 1 μg) constructs, respectively. GFP and α-tubulin were used as the transfection and loading controls, respectively.

Next, we tested whether RHBDD1 could cleave proTGFα in a cell-based secretion assay. To better examine the cleavage and secretion of proTGFα, we incorporated a FLAG tag between the signal peptide and the EGF-like domain in the proTGFα protein (Fig. 4a). The metalloprotease inhibitor BB94 was used to inhibit the cleavage of proTGFα by metalloproteases. As shown in Fig. 4b, RHBDD1 cleaved proTGFα-FLAG, leading to its secretion into the culture medium in a dose-dependent manner. However, catalytically inactive RHBDD1 or RHBDL2, a mammalian rhomboid protease for which several substrates have been identified^13^,^19^,^20^, was unable to trigger proTGFα cleavage and secretion (Fig. 4c). We further examined the RHBDD1-dependent secretion of endogenous TGFα in the presence of TACE by enzyme-linked immunosorbent assay (ELISA). As shown in Fig. 4d, despite a slight increase of TACE activity, soluble TGFα secretion decreased by ∼50% in RHBDD1-mutant HCT116 cells when compared with wild-type cells, whereas the secretion of EGF (the substrate for RHBDL2), TNFα (the substrate for TACE) and other EGFR ligands (HB-EGF, AREG, BTC and EPR, Supplementary Fig. 5f) did not change significantly. Furthermore, the reintroduction of RHBDD1 recombinant protein in catalytically inactive HCT116 cells significantly increased soluble TGFα secretion (Fig. 4e).

It has been reported that rhomboid intramembrane proteases recognize similar substrate motifs and the identification of this recognition motif made it feasible to predict the substrate cleavage site^21^,^22^. Therefore, previously reported recognition motifs in TatA, Gurken, Lac YTM2, spitz and EGF were aligned with proTGFα. The alignment result indicated that proTGFα cleavage most likely occurs at 93A–94S site in the juxtamembrane region between Ala-89 and Ala-102 (Fig. 4f). We first prepared proTGFα deletion construct (TGFα-Δ89–102) and performed the secretion assay to verify the predicted cleavage region. The result showed that RHBDD1 was unable to trigger the secretion of proTGFα-Δ89–102 in the presence of BB94. We next mutated proTGFα in the predicted critical positions at P1 (A93F), P2' (Q95G), P4 (V90G) and P2'/P4 (Q95G/V90G), and then performed the secretion assay. The result showed that RHBDD1 could trigger the secretion of wild-type proTGFα but not proTGFα variants mutated in P1, P4 and P2'/P4 in the presence of BB94. Taken together, these data demonstrate that RHBDD1-cleaved proTGFα within the juxtamembrane region at Ala 93.

In virtue of the existing evidence that TACE mediated the cleavage of proTGFα in mammals^3^, we compared the effect of RHBDD1 and TACE on soluble TGFα secretion in the presence or absence of one another. First, we knocked down RHBDD1 and TACE, respectively, in HCT116 cells and observed the soluble TGFα secretion in these cells. Soluble TGFα secretion decreased by 55.7% in RHBDD1-knockdown cells and 27.0% in TACE-knockdown cells when compared with wild-type cells (Supplementary Fig. 6a). Then, we employed CRISPR/Cas9 mediated gene knockout technique to knock out RHBDD1 and TACE, respectively, in RKO cells and observed the soluble TGFα secretion in these cells. Soluble TGFα secretion decreased by 45.3% in RHBDD1-knockout cells and 35.3% in TACE-knockout cells when compared with wild-type cells (Supplementary Fig. 6b). We further observed how RHBDD1 activity contributed to cleaved proTGFα in the presence of TACE. The result showed that the cleaved and secreted proTGFα-FLAG augmented significantly with the increase of RHBDD1 transfection dose (Supplementary Fig. 6c), indicating that RHBDD1 activity played a significant role in proTGFα cleavage even in the presence of TACE.

### RHBDD1 triggers activation of the EGFR signalling pathway

**Figure 5:**
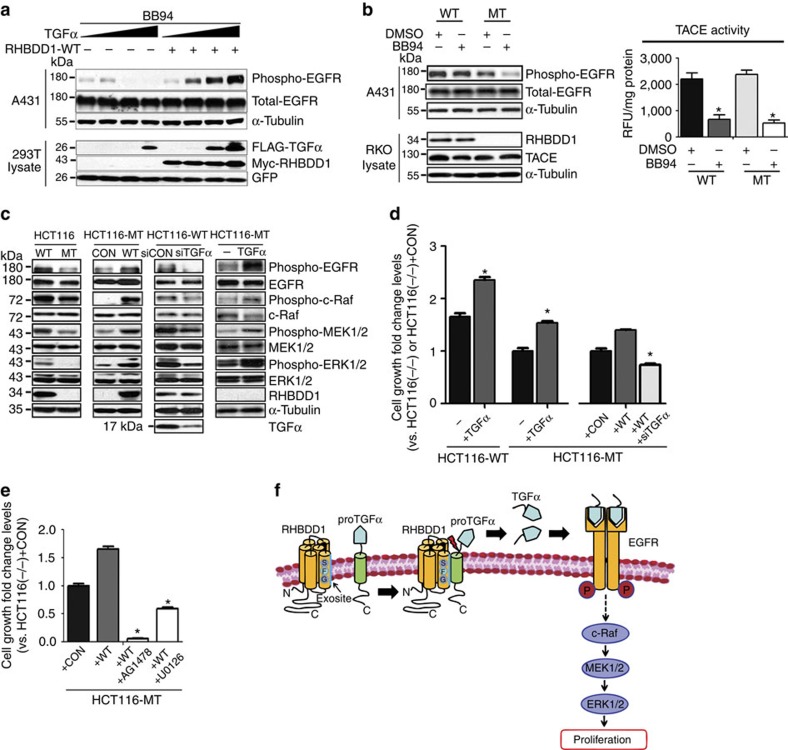
RHBDD1 triggers the activation of EGFR/Raf/MEK/ERK signalling pathway. (**a**) RHBDD1 drives EGFR activation. HEK 293T cells were transfected with proTGFα-FLAG (0, 250, 500 or 1,000 ng) and RHBDD1-Myc (500 ng) constructs, and cultured in serum-free medium containing 10 μM BB94. The culture supernatants were harvested, centrifuged and added to serum-starved A431 cells. The stimulated A431 cells were lysed and analysed by western blotting. α-tubulin and GFP were used as the loading and transfection controls, respectively. (**b**) Endogenous RHBDD1-cleaved proTGFα triggers EGFR activation. Culture supernatants were harvested from wild-type and RHBDD1-mutant RKO cells in the absence or presence of BB94, and then added to serum-starved A431 cells. The stimulated A431 cells were lysed and analysed by western blotting. α-tubulin was used as a loading control. (**c**) RHBDD1 and TGFα triggers the activation of EGFR/Raf/MEK/ERK signalling pathway. western blotting was performed to examine the phosphorylation and total levels of EGFR, c-Raf, MEK1/2 and ERK1/2 in wild-type and RHBDD1-mutant HCT116 cells with different treatment. α-tubulin was used as a loading control. (**d**) and (**e**) The growth inhibition associated with RHBDD1 inactivation was due to decreased TGFα/EGFR signaling. Different groups of HCT116 cells were transfected with proTGFα siRNA or treated with EGFR kinase inhibitor AG1478 or ERK1/2 inhibitor U0126. After 48 h, cell growth was measured by CCK-8 assay, and the relative fold changes were plotted. The data are presented as means±s.d., *N*=3, the Student's two-tailed *t*-test, *P<0.05. (**f**) Model illustrating the mechanisms of RHBDD1-mediated colon cancer cell proliferation.

To further study the biological role of RHBDD1 in proTGFα cleavage, we examined whether RHBDD1-cleaved proTGFα could activate EGFR. The A431 cell line, which expresses EGFR at a high level, was used in the following EGFR activation assays. Serum-starved A431 cells were exposed to supernatants from HEK293T cells expressing exogenous RHBDD1 and proTGFα in the presence of BB94. As shown in Fig. 5a, when ADAMs were inhibited, RHBDD1-cleaved proTGFα could significantly activate EGFR. We then sought endogenous evidence of RHBDD1-triggered EGFR activation. Serum-starved A431 cells were exposed to supernatants from wild-type and RHBDD1-mutant RKO cells in the absence or presence of BB94. As shown in Fig. 5b, when ADAMs were inhibited, EGFR could be activated significantly by endogenous RHBDD1-cleaved proTGFα in wild-type cells but not in RHBDD1-mutant cells. The effects of RHBDD1 and TGFα on the activation of EGFR/Raf/MEK/ERK signalling pathway was next examined. Significant decreases in the phosphorylation levels of the EGFR, Raf, MEK1/2 and ERK1/2 proteins were detected in mutant HCT116 cells compared with wild-type cells, and the reintroduction of recombinant RHBDD1 protein in mutant HCT116 cells significantly increased the phosphorylation of these proteins (Fig. 5c). Meanwhile, knockdown of proTGFα in wild-type HCT116 cells significantly decreased the activation of EGFR, Raf, MEK1/2 and ERK1/2; on the contrary, exogenous addition of soluble TGFα in RHBDD1-mutant HCT116 cells significantly increased the activation of these proteins (Fig. 5c). The important role of TGFα in tumor cell growth was further confirmed by proliferation assay, colony formation assay and soft agar assay (Supplementary Fig. 7). We then sought direct evidence that the decreased tumor cell growth associated with RHBDD1 inactivation was due to decreased TGFα/EGFR signalling. As shown in Fig. 5d, exogenous addition of soluble TGFα to mutant HCT116 cells significantly rescued the growth inhibition caused by RHBDD1 inactivation, and endogenous knockdown of proTGFα significantly decreased the growth acceleration caused by exogenous expression of RHBDD1 in mutant HCT116 cells. We further found that addition of either the EGFR inhibitor AG1478 or the MEK1/2 inhibitor U0126 could dramatically inhibit the growth promoting effect caused by exogenous expression of RHBDD1 in mutant HCT116 cells (Fig. 5e). Taken together, our data indicate that RHBDD1 triggers the activation of EGFR/Raf/MEK/ERK signalling pathway via the ADAM-independent secretion of TGFα.

### RHBDD1 correlates with EGFR activation in murine CRC

**Figure 6:**
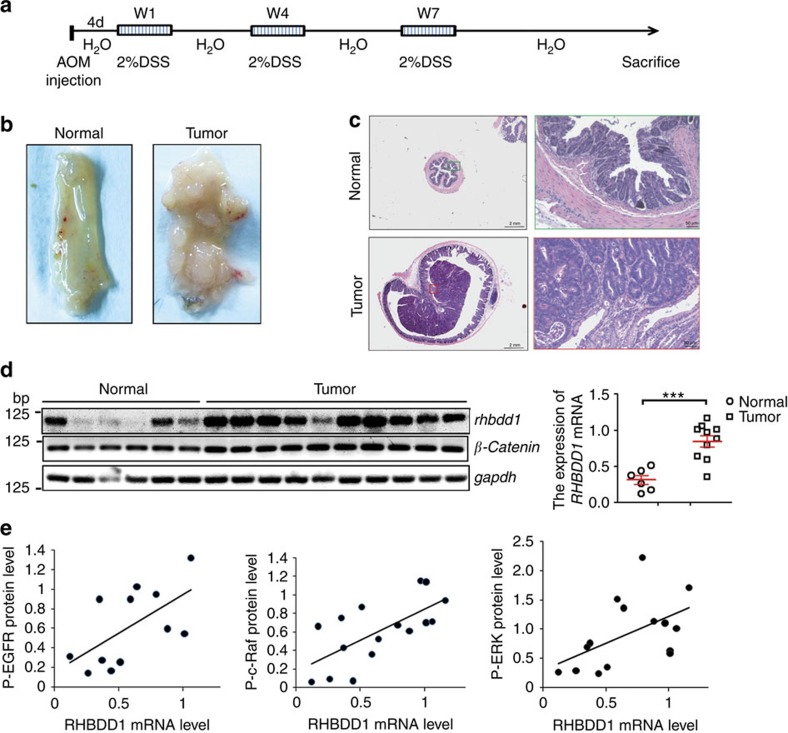
RHBDD1 expression in a murine model of colitis-associated colorectal cancer and its correlation with the EGFR signalling pathway. (**a**) Schematic representation of AOM and DSS treatment. (**b**) Representative gross appearance of the colons of normal and AOM/DSS-treated mice. (**c**) Representative images of colon tissues stained with H&E. (**d**) RHBDD1 is upregulated in the murine model of colorectal cancer. RHBDD1 and β-catenin mRNA expression was analysed by RT–PCR using GAPDH as a loading control. The data are presented as means±s.d., *N*=16, the Student's two-tailed *t*-test, ***P<0.001. (**e**) The level of RHBDD1 mRNA expression is positively correlated with that of phosphorylated EGFR, c-Raf and ERK1/2. The phosphorylation levels of EGFR, c-Raf and ERK1/2 were analysed by western blotting using GAPDH as a loading control. The relative level of RHBDD1 expression was plotted against the phosphorylation levels of EGFR, c-Raf or ERK1/2.

To support the role of RHBDD1 in CRC and to substantiate the functional link between RHBDD1 and the EGFR signalling pathway, we performed confirmatory experiments in a murine model of colitis-associated CRC. Accordingly, C57BL/6 mice were treated once with azoxymethane (AOM) followed by three cycles of orally administered dextran sulfate sodium (DSS) (Fig. 6a). We analysed sections of colorectal tissue harvested from mice at approximately day 80 and found that all the AOM/DSS-treated mice showed significant tumor development (Fig. 6b,c). The expression of *RHBDD1* mRNA in colorectal tumor tissue and control tissues was then determined by RT–PCR. The result revealed a statistically significant increase in *RHBDD1* expression in tumours compared with normal tissues (*N*=16, the Student's two-tailed *t*-test, *P*<0.001, Fig. 6d). Additionally, western blot analysis showed significant increases in the phosphorylation levels of EGFR, c-Raf and ERK1/2 in tumours compared with normal tissues (Supplementary Fig. 8). Further statistical analysis revealed Spearman's correlation coefficients of 0.609, 0.64 and 0.577 and Pearson correlation's coefficients of 0.604, 0.673 and 0.543 when the relative level of *RHBDD1* expression was plotted against the phosphorylation levels of EGFR, c-Raf and ERK1/2, respectively, indicating a significant positive correlation between RHBDD1 and the EGFR/Raf/ERK signalling pathway (Fig. 6e).

## Discussion

Regulated intramembrane proteolysis is a widely conserved and precise mechanism for controlling diverse biological processes^23^. To date, four protease families have been implicated in regulated intramembrane proteolysis: the presenilin, site 2 protease, signal-peptide peptidase and rhomboid families^24^,^25^. Rhomboid enzymes were originally identified using classical developmental genetics in *Drosophila*, and since then, significant progress has been made in understanding their functions^8^,^26^,^27^,^28^. Recent work has uncovered a role for a rhomboid member, presenilin-associated rhomboid-like protein, in Parkinson's disease^29^,^30^,^31^, which implies that rhomboid proteins are involved in human disease. Our previous work uncovered a new member of the rhomboid family, RHBDD1, and demonstrated that the biological role of this protease is to cleave different substrates^32^,^33^,^34^. In the present study, we provide new clinical evidence that RHBDD1 shows a significantly higher expression in human CRC samples and that its high expression is closely associated with poor CRC patient survival. The result that higher RHBDD1 expression was closely related to lower differentiation may imply a potential relationship between RHBDD1 and tumor growth. Generally, tumours with low differentiation grow faster than tumours with high differentiation. Therefore, the correlation between RHBDD1 expression and tumor differentiation supports our study on the growth-promoting effect of RHBDD1 in tumor cells. Moreover, the result that RHBDD1 expression was positively correlated with pTNM and pN classification indicates a potential correlation between RHBDD1 and malignancies with lymph node metastasis. Because the majority of the patients enroled in our study were patients with stage II/III colorectal carcinoma, a clinical investigation in patients with stage IV colorectal carcinoma is required to affirm the relationship between RHBDD1 and tumor metastasis, and a further study is needed if that is the case. Overall, our clinical findings demonstrate for the first time that RHBDD1 is closely related to the occurrence and progression of human CRC, and meanwhile indicate that RHBDD1 may be a useful biomarker for poor prognosis in CRC.

Our previous study showed that the exosomes secreted from RHBDD1-mutant cells could induce Jurkat cell apoptosis, and that the anti-apoptotic effect of RHBDD1 occurred under different apoptotic stimuli but not under normal condition^32^,^34^. In this study, we also had not detected any apoptosis in RHBDD1-mutant HCT116 and RKO cells and even in the tumours derived from these mutant cells. However, we found that RHBDD1 inactivation significantly inhibited tumor cell growth *in vitro* and *in vivo* and that this inhibition could largely be attributed to the RHBDD1-mediated positive regulation of cell proliferation. Therefore, the mechanism for RHBDD1 in promoting tumor cell growth was further explored. Eukaryotic cells are known to contain many different subcellular environments, and it is important to determine the subcellular localization of a protein to understand its function. In our study, we used somatic cell knock-in methods to accurately identify the subcellular localization of endogenous RHBDD1. We found that RHBDD1 was mainly localized in the filopodial-like projections in plasma membrane but distributed equally in cytoplasm of HCT116 cells. More importantly, the plasma membrane localization suggests a potential role for RHBDD1 in tumor cell growth that differs from our previous findings.

A currently accepted model for rhomboid substrate recognition involves the binding of the transmembrane domain (TMD) of the rhomboid substrate to the intramembrane ‘exosite' on the rhomboid enzyme, followed by the binding of the substrate recognition motif to the solvent-exposed rhomboid active site region^22^. We consequently utilized a two-step process to validate the cleavage of proTGFα by RHBDD1. First, we verified the specific interaction between RHBDD1 and proTGFα using co-immunoprecipitation assay and GST pull-down assay. Then, we examined the secretion of soluble TGFα induced by RHBDD1 through cell-based secretion assay and ELISA. The substrate specificity of RHBDD1 was further confirmed by showing that RHBDD1 neither interacted with other EGFR ligands, including EGF, HB-EGF, AREG, BTC and EPR, nor did it induce the secretion of these ligands. Interestingly, we noted that RHBDD1 could not only induce the cleavage and secretion of proTGFα, but also affect the steady level of this protein, which raised the possibility that the increase in proTGFα level resulted in the increased level of secreted TGFα. We excluded this possibility by experimentally showing that RHBDD1 could stabilize either wild-type or mutant proTGFα level, but only wild-type proTGFα could be cleaved and secreted into medium, demonstrating that RHBDD1-induced TGFα secretion had little to do with the steady level of proTGFα affected by RHBDD1. Taken together, our results show for the first time that RHBDD1 specifically triggers proTGFα secretion in a metalloprotease-independent manner.

Genetic analysis in *Drosophila* led to the discovery that the rhomboid intramembrane serine proteases control EGFR signalling. In *Drosophila*, rhomboids provide the proteolytic activity needed to release Spitz and two other TGFα-like ligands to activate EGFR^35^,^36^,^37^. Previous evidence indicates that the metalloproteinase TACE is the principal sheddase for proTGFα and other EGF ligands in mammals^12^,^38^,^39^. Over the years, no compelling evidence has been provided to either support or refute the idea that mammalian rhomboids participate in EGFR signalling. However, the recent discovery that EGF is an efficient substrate of the mammalian rhomboid RHBDL2 provides new and direct evidence to support this idea^13^. In the present study, we provide novel evidence that RHBDD1 induces the specific cleavage and ADAM-independent secretion of proTGFα and therefore activates the EGFR/Raf/MEK/ERK signalling pathway. This finding further supports the view that mammalian rhomboids are involved in EGFR signalling. On the basis of both the experimental data provided here and previously published results, we proposed a model for the growth-promoting effect of RHBDD1. Initially, the intramembrane ‘exosite' on RHBDD1 recognizes and recruits proTGFα. Subsequently, the recognition motif of proTGFα gains access to the active site on RHBDD1, and proTGFα is cleaved and released into the extracellular space. Finally, the secreted TGFα activates the EGFR/Raf/MEK/ERK signalling pathway, enhancing tumor cell proliferation (Fig. 5f). Our work provides the first mechanistic evidence of the involvement of mammalian rhomboid in the regulation of CRC cell growth.

The ability to proliferate independent of signals from other cell types is a fundamental characteristic of tumor cells^40^, and autocrine TGFα is an important determinant of abnormal growth in malignant progression. However, previous findings indicating that normal cells also display autocrine TGFα activity^41^,^42^ raise the question of how this autocrine growth factor imparts a growth advantage to tumor cells that is not shared with normal cells. On the basis of our data, we speculate that one mechanism may involve proTGFα activation via the high expression of RHBDD1 in tumours. The finding that proTGFα could be cleaved and activated by RHBDD1 in a dose-dependent manner in the presence of TACE further supports the idea that the increased RHBDD1 expression promotes the abnormal tumor cell growth. The only genetic evidence implicating a protease in TGFα secretion was obtained using TACE−/− embryonic fibroblasts^3^,^12^; however, it is currently unclear whether this protease has such a role in colorectal tissue. In our study, the comparative results indicated that TACE and RHBDD1 could both individually drive proTGFα cleavage and secretion in cancer cells. Therefore, we infer that the differential expression patterns of RHBDD1 and TACE in different cells might account for their parallel regulation of EGFR signalling. However, the tumor-restricted expression pattern of RHBDD1 renders this protease more important in tumorigenesis when compared with TACE which is widely expressed in tissues. Moreover, the generation of RHBDD1-knockout mice will provide additional genetic evidence for the role of RHBDD1 in tumorigenesis.

In summary, our current findings demonstrate a novel role for RHBDD1 in promoting tumor cell growth through the ADAM-independent cleavage and secretion of proTGFα to activate the EGFR/Raf/MEK/ERK signalling pathway in CRC. Further studies will address whether RHBDD1 plays a similar role in other tumours. In addition, our data show that RHBDD1 could be a potential prognostic biomarker or therapeutic target for CRC, and further investigation is required to achieve its clinical application.

## Methods

### Cell culture and reagents

HCT116, RKO, HT29, DLD1, LOVO and SW620 cells were cultured in Iscove's Modified Dulbecco's Medium (HyClone) with 10% fetal bovine serum (FBS), and HEK293T cells were cultured in Dulbecco's Modified Eagle Medium (HyClone) with 10% FBS. All cell lines were obtained from the Cell Resource Center of Peking Union Medical College. Plasmids were constructed according to standard cloning techniques. Expression plasmids for RHBDD1 and RHBDL2 were cloned into pcDNA6.0 with a C-terminal Myc tag, and expression plasmids for EGFR ligands (proTGFα, EGF, HB-EGF, AREG, BTC and EPR) were cloned into pcDNA6.0 with a C-terminal V5 tag. A plasmid expressing proTGFα with a FLAG tag in the pro-peptide (between residues 33 and 34) was generated by splice overlap extension-PCR^43^. RHBDD1 and proTGFα point mutants were generated using the Quikchange Lightning Site-Directed Mutagenesis Kit (Agilent Technologies) according to the manufacturer's instructions. An Alexa Fluor 594 conjugate of WGA was purchased from Invitrogen, Batimastat (BB94) was purchased from Thermo Scientific, MG132 was purchased from Sigma-Aldrich, Bortezomib (Velcade) was purchased from Beijing Chief-East Tech Co., Ltd., AG1478 was purchased from Selleck Chemicals and U0126 was purchased from Cell Signaling Technology. All compounds were dissolved in DMSO to a final concentration of 10 mmol l^−l^ and stored at −20 °C.

### Antibodies

The anti-RHBDD1 mouse monoclonal antibody was prepared in-house. Antibodies against Na^+^/K^+^-ATPase, TACE, phospho-c-Raf, c-Raf, phospho-MEK1/2, MEK1/2, phospho-ERK1/2, ERK1/2, phospho-EGFR, EGFR and α-tubulin were purchased from Cell Signaling Technology. Antibodies against FLAG, Myc, V5 and GST were purchased from Sigma-Aldrich. proTGFα, β-actin, GAPDH and GFP antibodies were purchased from Santa Cruz Biotechnology, Inc.

### Tissue analyses

The tissue microarray including tumor tissues and their corresponding adjacent normal tissues from 142 cases of CRC was obtained from Shanghai Biochip; the tissue microarray including tumor tissues from 539 cases of CRC was conducted on paraffin-embedded tumor samples, which were histopathologically diagnosed at the Cancer Hospital, Chinese Academy of Medical Sciences, from January 2005 to December 2008. Prior patient consent and approval from the Institutional Research Ethics Committee were obtained for the use of these clinical materials for research purposes. The clinical information regarding the samples was collected and summarized in Table 1 and Supplementary Table 1. Paraffin-embedded tissue sections (4 μm) were prepared according to classic methods, and the expression of RHBDD1 (1:200 dilution) was detected using immunoperoxidase. Slides were assessed by pathologists who did not have knowledge of experimental results or patient outcome. The RHBDD1 expression was evaluated by immunostaining score, which was calculated as the sum of the proportion and intensity of the stain^44^. Briefly, a proportion score was assigned first, which represented the estimated proportion of positive-staining tumor cells (0, none; 1, <1/100; 2, >1/100 to <1/10; 3, >1/10 to <1/3; 4, >1/3 to <2/3; and 5, >2/3). Next, an intensity score was assigned, which represented the average intensity of positive tumor cells (0, none; 1, weak, 2, intermediate; and 3, strong). The proportion and intensity scores were then added to obtain a total score, which ranged from 0 to 8. Immunohistochemical staining of Ki67 and proliferating cell nuclear antigen were conducted using polyclonal antibodies from Cell Signaling Technology.

### Targeted knock-in of RHBDD1-mutants

Targeted knock-in of RHBDD1 mutants was performed by improved somatic cell knock-in method as described previously^45^. Briefly, targeting constructs for mutation of RHBDD1 (G142A and S144A) were generated by cloning 0.9- and 1.1-kb RHBDD1 genomic DNA fragments surrounding exon 2 of the RHBDD1 gene, respectively, into the bipartite targeting vectors depicted in Supplementary Fig. 3a. HCT116 and RKO colon cancer cells, which contain two intact alleles of RHBDD1, were infected with the AAVs containing the targeting constructs, and the cells were selected in G418 (0.5 mg ml^−1^) for 2 weeks. Neomycin-resistant clones were then screened for targeting events by PCR by using primers for RHBDD1 and the neomycin-resistance gene. The targeting events were confirmed by PCR using different primer pairs (Supplementary Table 3). The DNA fragments from the confirmatory PCRs were then sequenced to ensure the presence of the mutant alleles. To target the second allele of RHBDD1 with the same targeting virus, correctly targeted clones were infected with an adenovirus expressing the Cre recombinase to delete the neomycin-resistance gene. To select clones with successful deletion of the neomycin-resistance gene, PCR were carried out to amplify an ∼200-bp genomic fragment in which the Lox P site was inserted. The heterozygous knock-in clones were infected with the same targeting virus to target the second allele and the neomycin-resistance gene was excised as described earlier. Finally, the homogenous knock-in clones of RHBDD1 mutation were verified by sequencing, RT–PCR and western blotting.

### RT–PCR and real-time PCR

Total RNA was isolated from different cell lines using TRIZOL Reagent (Invitrogen) according to the manufacturer's instructions. Equal amounts of RNA were reverse transcribed into cDNA using the ReverTra Ace-α-First strand cDNA synthesis kit (Toyobo) as instructed by the manufacturer. Quantitative PCR was performed using the ABI StepOne Plus system. PCR reactions were carried out in 10 μl reactions using SYBR Green PCR master mix (Invitrogen) and 0.5 μM specific primers. The primers used for PCR are shown in Supplementary Table 3.

### Cell proliferation related assays

Cell proliferation was assessed using the CCK-8 assay (Dojindo Molecular Technologies). Briefly, HCT116 and RKO cells were seeded in 96-well plates. Ten microliters of CCK-8 solution was added to each well containing 100 μl culture medium and incubated for 2 h at 37 °C. The absorbance was measured at 450 nm using an ELISA plate reader. For cell proliferation assays, cell growth was measured once per day for 6 days; for growth inhibition assays, cell growth was measured 2 days after transfected with proTGFα siRNA or treated with EGFR kinase inhibitor AG1478 or MEK1/2 inhibitor U0126.

### Soft agar colony formation assays

Cells were mixed with cell culture medium containing 0.6% agar to obtain a final concentration of 0.3%. Two millilitres of the cell suspension, which contained 1 × 10^4^ cells, was immediately plated in six-well plates coated with cell culture medium containing 0.6% agar (2 ml per well). Two weeks after plating, the colonies were counted in triplicate.

### *In vivo* tumorigenesis

Animal experiments were performed with the approval of Peking Union Medical College animal care and use committees. Five million tumor cells were resuspended in 0.2 ml phosphate-buffered saline and inoculated into the flanks of 6-week old, female, athymic nude mice. Five mice were injected in each group. Tumor growth was monitored every 3 days by measuring tumor diameters. Tumor width (W) and length (L) were measured, and tumor volume was calculated using the following formula: volume=(W × L)^2^/2. Mice were sacrificed 23 days after inoculation. Tumours were removed, photographed and weighed and the average weights of the tumours were calculated (*P<0.01).

### Lentivirus-mediated transduction

For the expression of human RHBDD1, the Myc-tagged gene was cloned into the target vector pIRES2-EGFP and then subcloned into the expression vector plenti6.3/V5-DESTs. ViraPowerLentiviral Expression Systems (Invitrogen) were used to produce lentivirus. Briefly, 293FT cells were transfected in 10-cm plates with 3 μg of the expression plasmid and 9 μg of the ViraPowerPackaging Mix. The following day, the medium was replaced and the transfected cells were allowed to secrete virus for 48 h. Culture supernatants were then filtered using 0.45 μm filters. For the infection of target cells, the viral supernatants were diluted fourfold in fresh medium before transduction. Transduction was carried out in the presence of 5 μg ml^−1^ polybrene, and the medium was changed 12 h later. To isolate the cells that expressed GFP, cells were dissociated into a single-cell suspension using trypsin. Before sorting, aggregates were removed by passing the cells through a 40-μm cell strainer. FACS was performed using a BD Aria II sorter, which was gated for a moderate level of GFP expression. For the knockdown of human RHBDD1, RHBDD1-specific shRNAs (shRNA1: GTAGATGGTTTGCCTATGT, shRNA2: GGATTCTTGTTGGACTAAT) were packaged into lentivirus from Genechem Inc.

### Endogenous RHBDD1 epitope tagging

FLAG and SBP tagging of the endogenous RHBDD1 gene was performed by improved somatic cell knock-in method as described previously^46^. Briefly, a PCR fragment ∼1kb extending from intron to the last exon before the stop codon was amplified from genomic DNA and cloned in-frame with the FLAG-SBP tag sequence as the left homologous arm. A PCR fragment from the sequence after the TGA stop codon extending to the 3′ end of the targeted genes was also amplified from genomic DNA to be used as the right arm (Supplementary Fig. 3c). Primers used to amplify homologous arms and confirm the targeting events were shown in Supplementary Table 3.

### Immunofluorescence staining

Living cells were incubated with Alexa Fluor 594 WGA for 10 min and then washed with PBS. The cells were fixed with 4% formaldehyde and permeabilized with PBS containing 0.5% Triton X-100. The slides were blocked with 5% BSA and incubated with anti-FLAG antibody at 4 °C overnight. A FITC-conjugated secondary antibody was added for 30 min at 37 °C. The slides were stained with DAPI, mounted and observed under a microscope. As a negative control, the specific primary antibodies were replaced with a control mouse IgG.

### Isolation of plasma membrane proteins

For cell membrane isolation assay, plasma membrane extracts were prepared using a plasma membrane protein extraction kit (BioVision). Extraction of plasma membrane and cytosolic proteins were performed according to the manufacturer's instructions. For cell surface protein isolation assay, cell surface proteins were biotinylated and isolated using a Pierce cell surface protein isolation kit (Thermo Scientific). Isolation of cell surface proteins was performed according to the manufacturer's instructions. Each fraction was tested for the presence of plasma membrane marker Na^+^/K^+^-ATPase and cytosolic marker GAPDH by western blotting.

### Affinity purification and mass spectrometry (MS)

The targeted knock-in cells were lysed in lysis buffer (50 mM Tris-HCl (pH 7.4), 100 mM NaCl, 0.5% NP-40, 1 mM EDTA and protease inhibitor cocktails) and subjected to affinity purification with anti-FLAG M2 affinity gel. The purified protein complex was resolved on SDS—PAGE and silver stained. The differential protein bands was excised manually from the gel slab, cut into pieces and put into 1.5-ml Eppendorf vials. Sample was in-gel reduced and alkylated and then dehydrated by acetonitrile (ACN). Gel pieces were further rehydrated and digested by trypsin using overnight method. The digested peptides were extracted from the gel by ACN, and lyophilized. The digested samples were analysed by RP C18 capillary liquid chromatography (LC) column from MichromBioresources (100 μm × 150 mm, 3 μm). The eluted gradient was 5–30% buffer B1 (0.1% formic acid, 99.9% ACN; flow rate, 0.5 μl min^−1^) for 30 min. The eluted peptides were analysed by LTQ OrbitrapVelos (Thermofisher). The MS data were acquired using the following parameters: 20 data-dependent collision-induced dissociation (CID) MS/MS scans per every full scan; full scans was acquired in Orbitrap at resolution 60,000; 35% normalized collision energy in CID included internal mass calibration (445.120025 ion as lock mass with a target lock mass abundance of 0%), charge state screening (excluding precursors with unknown charge state or +1 charge state) and dynamic exclusion (exclusion size list 500, exclusion duration 30 s). The MS/MS spectra were respectively searched against the SwissProt human database from Uniprot website ( www.uniprot.org) using the Bioworks (version 3.3.1 sp1, Thermofisher). Trypsin was chosen as cleavage specificity with a maximum number of allowed missed cleavages of two. Carbamidomethylation (C) was set as a fixed modification. The searches were performed using a peptide tolerance of 5 p.p.m. and a product ion tolerance of 0.5 Da. The results were further analysed by following filter (1) DeltaCn score is at least 0.1; (2) Rsp score is 1; (3) Xcorr. 2.8, 3.5 for +2, +3 charged peptides.

### Immunoprecipitation assay

Immunoprecipitation assays were performed as previously described^47^. For co-immunoprecipitation assays, HEK293T cells were transiently transfected with pcDNA6-V5-proTGFα and pcDNA6-Myc-RHBDD1-wt or pcDNA6-Myc-RHBDD1-mt plasmids as indicated. After a 48 h incubation, cells were harvested and lysed in lysis buffer (50 mM Tris-HCl (pH 7.4), 150 mM NaCl, 1% NP-40, 1 mM EDTA, 10% glycerol and protease inhibitor cocktails). Solubilized proteins were isolated using anti-V5 or anti-Myc affinity gel, respectively. Immunocomplexes were solubilized in 1.25 × SDS loading buffer and immunoblotted with indicated antibodies. For immunoprecipitation assays, HCT116 cells were harvested and lysed in lysis buffer [50 mM Tris-HCl (pH 7.4), 100 mM NaCl, 0.5% NP-40, 1 mM EDTA and protease inhibitor cocktails]. Solubilized proteins were isolated using anti-FLAG M2 affinity gel (Sigma-Aldrich). Immunocomplexes were solubilized in 1.25 × SDS loading buffer and immunoblotted with indicated antibodies.

### GST pull-down assay

GST pull-down assays were performed as previously described^47^. Briefly, 400 μg of HCT116 cell lysates (50 mM Tris-HCl (pH7.4), 150 mM NaCl, 1% NP-40, 1 mM EDTA and protease inhibitor cocktails) mixed with 2 μg of purified GST-proTGFα or GST, immobilized on glutathione-Sepharose beads for 2 h at 4 °C and washed three times with 1 × PBS. Next, the GST pull-down products were run in a 12% SDS–PAGE gel and immunoblotted with anti-RHBDD1 and anti-GST antibodies.

### Rhomboid-based secretion assays

HEK 293T cells were transfected in 6-cm plates using Entranster-H transfection reagent (Engreen Biosystem Co., Ltd.) and the indicated proTGFα and RHBDD1 expression plasmids. The total amount of DNA was adjusted to 2 μg with the pcDNA6.0 vector. After transfection for 18 h, the cells were washed twice in PBS and the secretion assays were performed for 30 h in serum-free medium containing 10 μM BB94 or DMSO as a vehicle control. The culture supernatants were collected by centrifugation at 16,000*g* for 10 min, and the proteins were precipitated by adding TCA (12% w/v). The precipitated proteins were recovered by centrifugation, washed twice in acetone and solubilized in 30 μl 1 × SDS–PAGE loading buffer. In addition, the HEK293T cells were lysed in 300 μl of sample buffer. The samples were analysed by western blotting using the anti-FLAG and anti-Myc antibodies.

### Enzyme-linked immunosorbent assays

Tumor cell media was collected and analysed for soluble TGFα, EGF, TNFα, HB-EGF, AREG, BTC and EPR using an ELISA kit (Neobioscience Technology Company) according to the manufacturer's instructions. For normalization, the cells were trypsinized and the live cell fraction was counted using a handheld automated cell counter (Millipore Corporation).

### TACE activity assay

Culture supernatants were harvested from wild-type and RHBDD1-mutant HCT116 and RKO cells in the absence or presence of BB94. The intracellular TACE activity was performed according to the protocol of an InnoZyme TACE activity kit (Calbiochem).

### EGFR activation assays

HEK 293T cells were transfected in 6-cm plates with the indicated proTGFα and RHBDD1 expression plasmids. The following day, the cells were washed twice in PBS and then cultured in serum-free medium containing 10 μM BB94. After 36 h, the culture supernatants were harvested, centrifuged to remove cell debris and added to a sub-confluent monolayer of A431 cells, which had previously been serum starved for 24 h. Fifteen minutes later, the supernatants were aspirated off and the stimulated A431 cells were lysed in 1 × cell lysis buffer (Cell Signaling Technology). The samples were analysed by western blotting using anti-phospho-EGFR and total EGFR antibodies.

### Induction of colitis-associated CRC in mice

Animal experiments were performed with the approval of Peking Union Medical College animal care and use committees. 8-week old, female, C57BL/6 mice were injected with 10 mg kg^−1^ of AOM intraperitoneally at the beginning of the experiment. After 4 days, 2% (w/v) DSS (molecular weight:36,000–50,000) was given in drinking water by oral administration over 7 days followed by 14 days period of recovery with normal water. This cycle was repeated two times and mice were sacrificed 3 weeks after the last DSS cycle.

### Statistical analysis

The comparison of RHBDD1 expression in CRC and adjacent normal tissues was analysed by the Wilcoxon signed rank test. The evaluation of relationship between RHBDD1 expression and clinicopathological parameters of 539 CRC patients was performed using a *χ*^2^ test. Disease-free and Overall survival were analysed by the Kaplan–Meier method with log-rank testing. For cytological research, values represented mean±s.d. of samples measured in triplicate, and each experiment was repeated three times. The significance of differences between experimental groups was analysed using the Student's two-tailed *t*-test. A 2-tailed *P*<0.05 was considered statistically significant. All the analyses were performed by SPSS and GraphPad Prism 5.0.

## Additional information

**How to cite this article:** Song, W. *et al.* Rhomboid domain containing 1 promotes colorectal cancer growth through activation of the EGFR signalling pathway. *Nat. Commun.* 6:8022 doi: 10.1038/ncomms9022 (2015).

## Supplementary Material

Supplementary Information Supplementary Figures 1-9 and Supplementary Tables 1-3

## Figures and Tables

**Table 1:** Association between RHBDD1 expression and clinicopathologic parameters in 539 colorectal cancer patients.

Clinicopathologic parameters	*N*	RHBDD1 expression		*P* value*
		Negative	Positive	
Gender				0.193
Male	327	182	145	
Female	212	130	82	
Age				0.080
≤60	286	176	110	
>60	253	136	117	
Smoking				0.313
Yes	75	39	36	
No	464	273	191	
Alcohol abuse				0.745
Yes	52	29	23	
No	487	283	204	
Family history†				0.399
Yes	57	30	27	
No	482	282	200	
Differentiation				0.006*
Low	98	44	54	
Moderate	398	237	161	
High	43	31	12	
Necrosis				0.667
Yes	23	12	11	
No	516	300	216	
Lymphatic vessel				
Yes	31	13	18	
No	508	299	209	
pTNM				0.0001*
I	1	0	1	
II	340	220	120	
III	198	92	106	
pT				0.408
T1	1	0	1	
T2	6	2	4	
T3	518	302	216	
T4	14	8	6	
pN				0.0002*
N0	341	220	121	
N1	134	61	73	
N2	64	31	33	

pTNM, pathological tumor-node-metastasis; pT, pathological tumor; pN, pathological node.

^*^The significance of RHBDD1 expression in clinicopathologic parameters was calculated by *χ*^2^ test and Fisher's exact test was used when over 25% cells had expected count <5.

^†^Family history indicates that the immediate family member of the patient has a history of digestive system tumours.
