# Supplementary material for: Rhomboid domain containing 1 promotes colorectal cancer growth through activation of the EGFR signalling pathway
Source: Nat Commun. 2015 Aug 24;6:8022. doi: 10.1038/ncomms9022 (PMC4560765; doi:10.1038/ncomms9022)
Supplement: Supplementary Information — Supplementary Figures 1-9 and Supplementary Tables 1-3 [file ncomms9022-s1.pdf]

Supplementary information

Supplementary figures

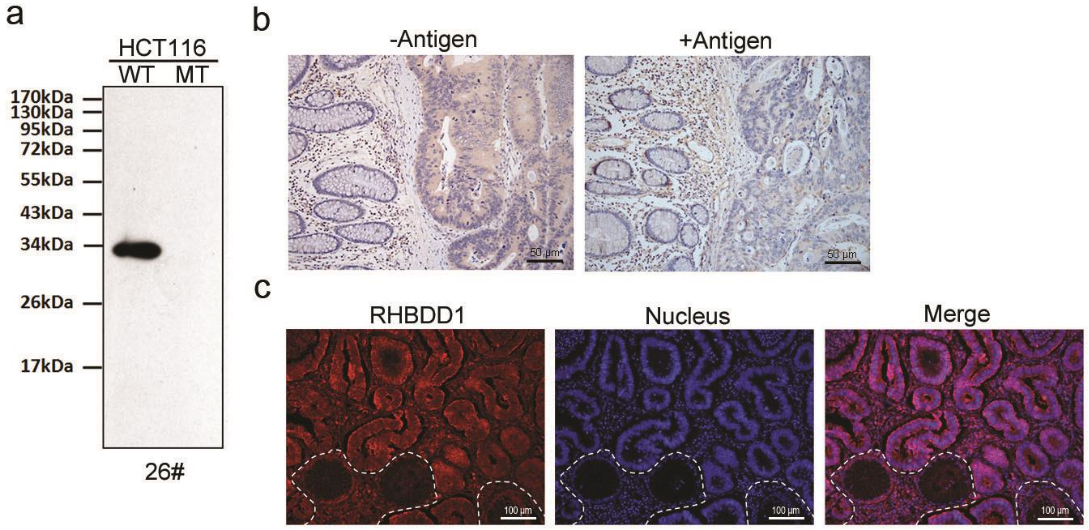

Supplementary Figure 1 | Determination of the specificity of in-house anti-RHBDD1 mouse monoclonal antibody. (**a**) The specificity of the anti-RHBDD1 monoclonal antibody was examined in wild-type and RHBDD1 mutant HCT116 cells by Western blotting. Antibody dilution: 1:5000. Sample loading: 20 µg. (**b**) The specificity of the anti-RHBDD1 monoclonal antibody was examined by immunohistochemical staining with absorption control. The tissue and the antibody were preincubated with purified RHBDD1 protein which was used to generate the antibody, and then immunohistochemical staining was performed according to the standard process. Antibody dilution: 1:200. (scale bar = 50 µm) (**c**) Immunofluorescence staining for RHBDD1 in paraffin sections of colorectal carcinoma. RHBDD1 expression was analyzed by immunofluorescence with anti-RHBDD1 monoclonal antibody (red). Cell nuclei were stained with DAPI (blue). Dashed lines represented the boundary between normal tissue and neoplastic tissue. Antibody dilution: 1:200. (scale bar = 100 µm).

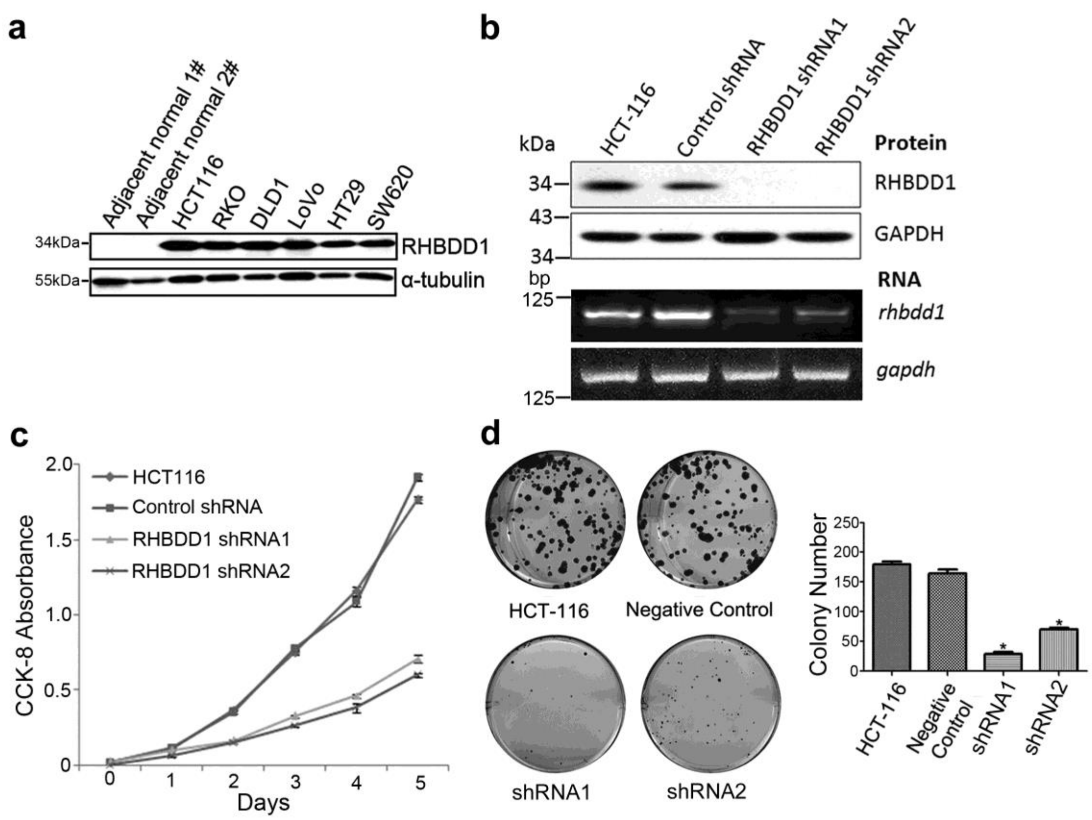

Supplementary Figure 2 | The effect of RHBDD1 knockdown on tumor cell growth. (**a**) The expression level of RHBDD1 in different human colon cancer cell lines was examined by Western blotting. α-tubulin was used as a loading control. (**b**) The efficiency of RHBDD1 knockdown in HCT116 cells was examined by Western blotting and RT-PCR. GAPDH was used as a loading control. (**c**) Cell proliferation assays. The samples were assayed in triplicate. Each point represents the mean value from 3 independent samples. (**d**) Colony formation assays. Representative photographs and the bar graph were from 3 independent experiments. The data are presented as means ± SDs, N = 3, the Student's 2-tailed t-test, * $P < 0.05$.

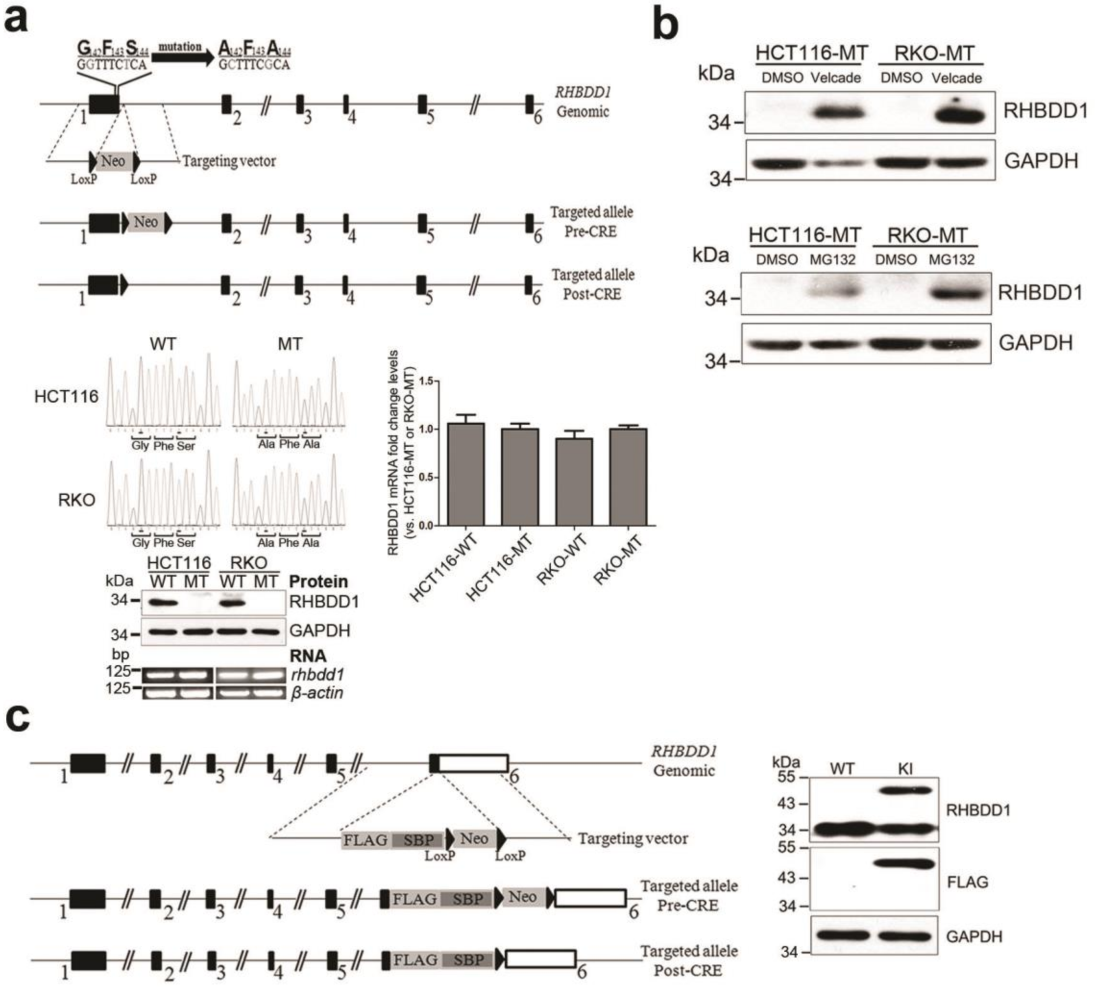

Supplementary Figure 3 | (**a**) Diagram of the RHBDD1 targeted mutation strategy. Boxes 1-6 represent the RHBDD1 coding sequences of exons 2-7. The targeting construct consisted of two homologous arms and the neomycin-resistance gene (Neo); the homologous left arm contained the mutations G142A and S144A. The targeted mutation of RHBDD1 in HCT116 and RKO cells with different genotypes was verified by genomic sequencing. The expression of RHBDD1 in HCT116 and RKO cells was examined in by Western blotting and RT-PCR. GAPDH and β-actin were used as loading controls. (**b**) Targeted mutation of RHBDD1 leads to its degradation through the proteasome pathway. Mutant HCT116 and RKO cells were treated with Velcade or MG132, and RHBDD1 was detected by Western blotting. GAPDH was used as a loading control. (**c**) Diagram of the RHBDD1 targeted knock-in strategy. Boxes 1-6 represent the RHBDD1 coding sequences of exons 2-7. The

targeting construct consisted of two homologous arms and a Neo-loxP-FLAG-SBP cassette. Homologous recombination resulted in the knock-in of the FLAG and SBP epitopes to the COOH-terminus of the RHBDD1 protein. Expression of wild-type and FLAG-tagged RHBDD1 was examined by Western blotting using anti-RHBDD1 and anti-FLAG antibodies. GAPDH was used as a loading control.

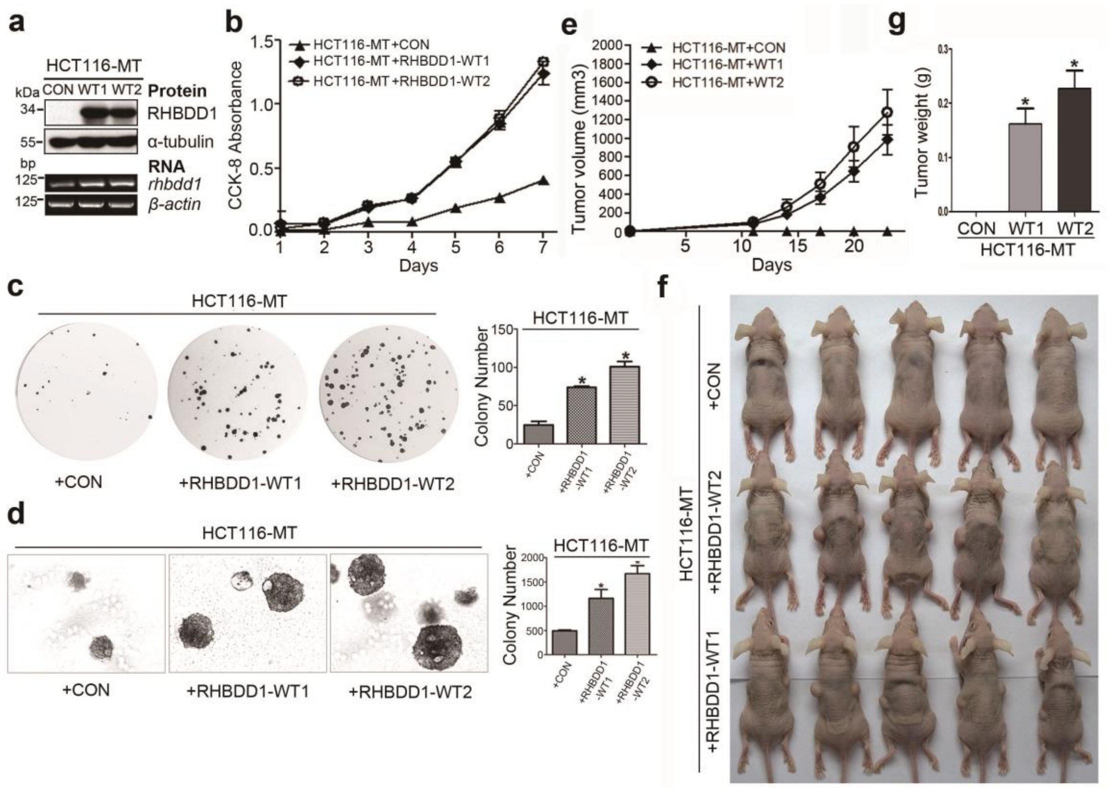

Supplementary Figure 4 | Tumor cell rescue experiments. (**a**) Expression of wild-type RHBDD1 recombinant proteins in mutant HCT116 cells was examined by Western blotting and RT-PCR. α-tubulin and β-actin were used as loading controls. (**b**) Cell proliferation assays. The samples were assayed in triplicate. Each point represents the mean value from three independent samples. (**c**) Colony formation assays and (**d**) Soft agar colony formation assays. Representative photographs and bar graphs are from three independent experiments. The data are presented as means ± SDs, N = 3, the Student's 2-tailed t-test, * $P < 0.05$. (**e**) Growth curves of xenograft tumors. Tumor volumes were monitored every three days by measuring tumor diameters. The data are presented as means ± SDs, N = 10. (**f**) and (**g**) Images and weights of xenograft tumors. The tumors were photographed, removed and weighed. The bar graphs represent means ± SDs, N = 10, the Student's 2-tailed t-test, * $P < 0.05$.

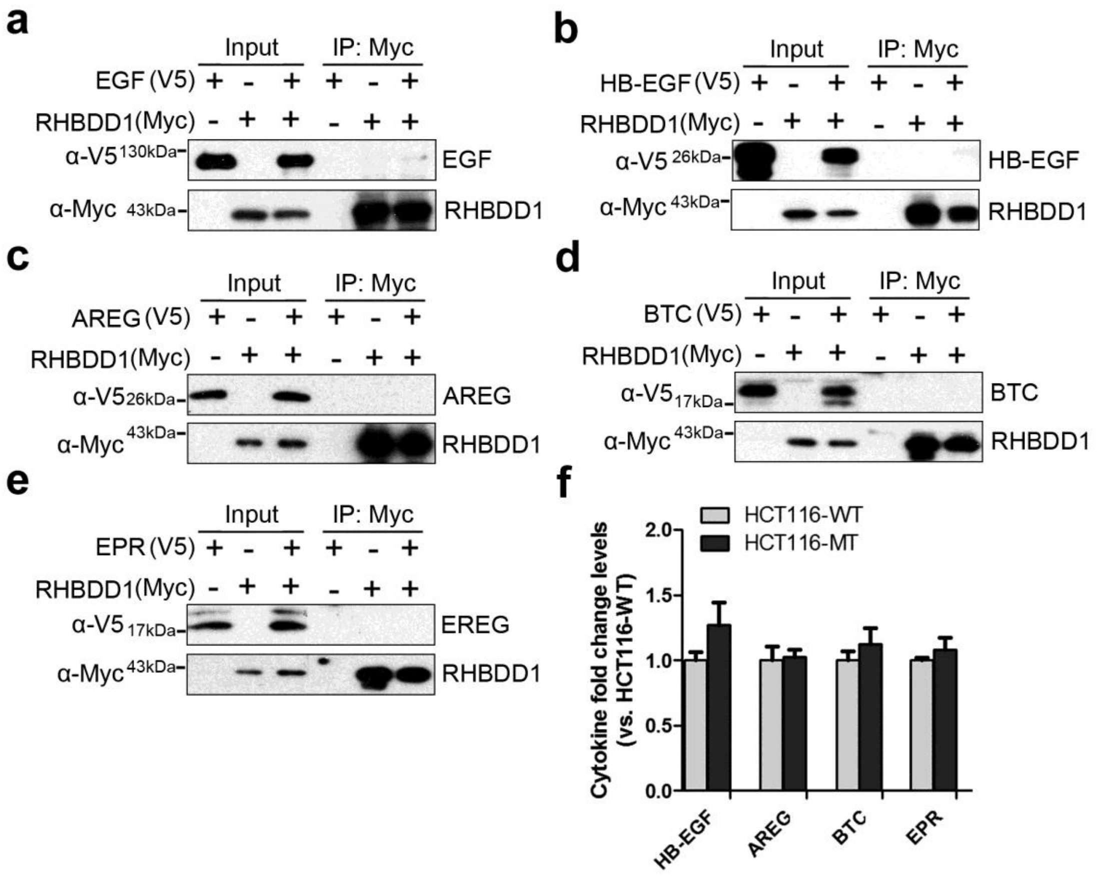

Supplementary Figure 5 | Determination of substrate specificity of RHBDD1. (**a-e**) V5-tagged EGFR ligands (EGF, HB-EGF, AREG, BTC and EPR) were respectively transfected into HEK 293T cells with Myc-tagged RHBDD1. After immunoprecipitation with the anti-Myc antibody, V5-tagged EGFR ligands were detected by Western blotting using the anti-V5 antibody. (**f**) Media from wild-type or mutant HCT116 cells were collected and assayed for EGF, HB-EGF, AREG, BTC and EPR levels by ELISA, and the relative fold changes were plotted.

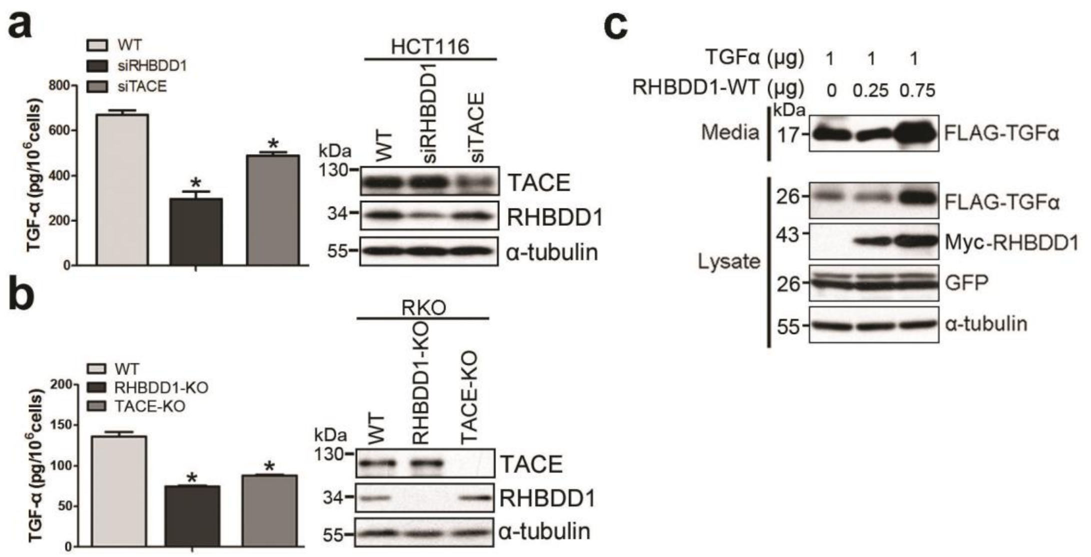

Supplementary Figure 6 | Comparative effects of RHBDD1 and TACE on TGFα cleavage and secretion. (**a**) and (**b**) Media from wild-type, RHBDD1-KD or TACE-KD HCT116 cells, and from wild-type, RHBDD1-KO or TACE-KO RKO cells were collected, and TGFα level was measured by ELISA. The data are presented as picograms per 10^6 cells ± SD of three independent experiments, N = 3, the Student's 2-tailed t-test, * $P < 0.05$. The expression of RHBDD1 and TACE in HCT116 and RKO cells was examined in by Western blotting. α-tubulin was used as a loading control. (**c**) HEK 293T cells were transfected with TGFα-FLAG (1 μg) and RHBDD1-Myc (0, 250 and 750 ng) constructs, and the secretion assay was performed in the presence of TACE. GFP and α-tubulin were used as the transfection and loading controls, respectively.

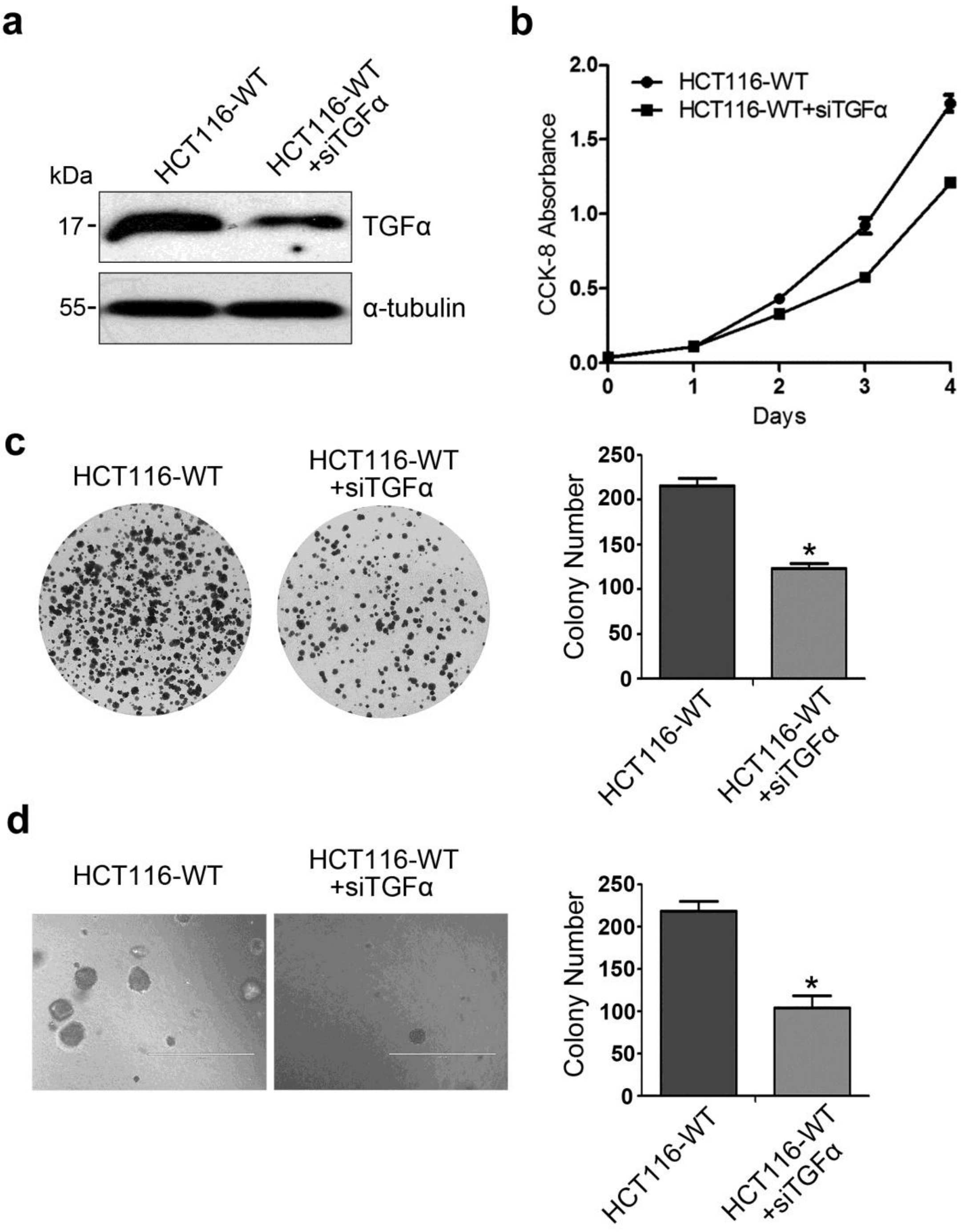

Supplementary Figure 7 | The effect of TGFα knockdown on tumor cell growth. (**a**) The efficiency of TGFα knockdown in HCT116 cells was examined by Western blotting. α-tubulin was used as a loading control. (**b**) Cell proliferation assays. The samples were assayed in triplicate. Each point represents the mean value from three independent samples. (**c**) Colony formation assays. Representative photographs and bar graphs are from three independent experiments. The data are

presented as means ± SDs, N = 3, the Student's 2-tailed t-test, * P < 0.05. (**d**) Soft agar colony formation assays. Representative photographs and bar graphs are from three independent experiments. The data are presented as means ± SDs, N = 3, the Student's 2-tailed t-test, * P < 0.05.

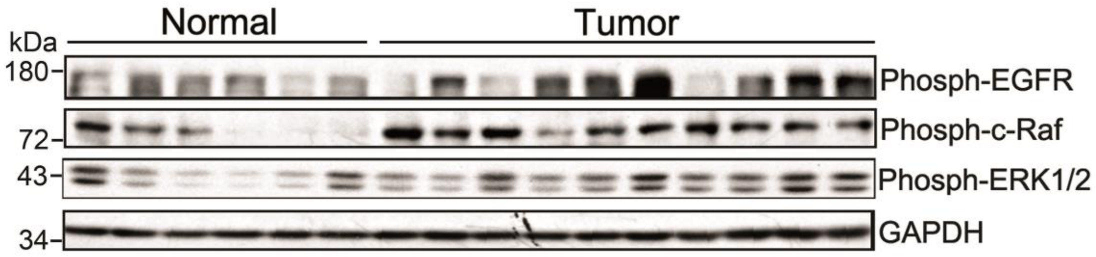

Supplementary Figure 8 | The phosphorylation levels of EGFR, c-Raf and ERK1/2 were examined in normal tissues and tumors from a murine model of colitis-associated colorectal cancer by Western blotting using the indicated antibodies. GAPDH was used as a loading control.

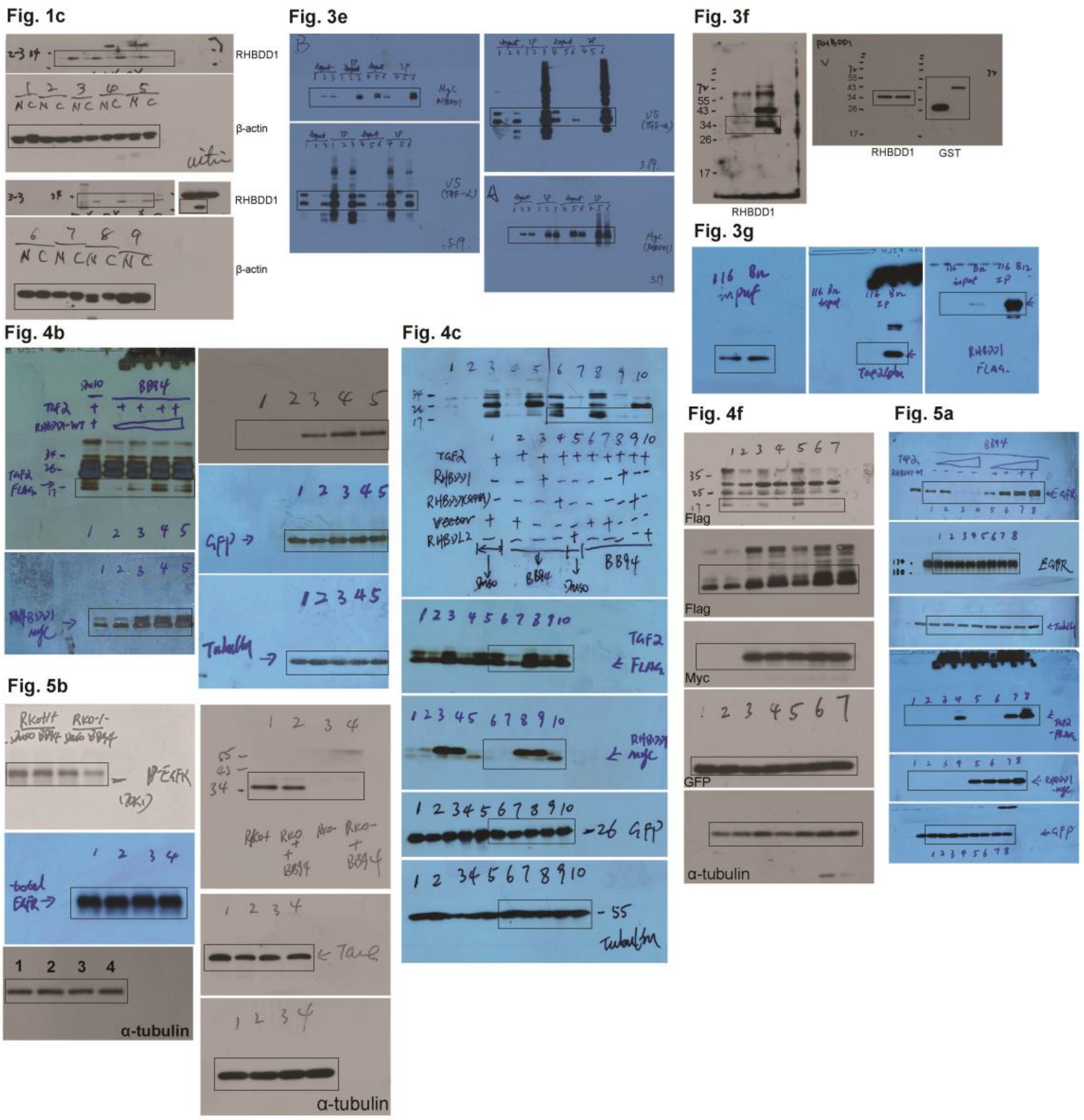

Supplementary Figure 9 | **Uncropped scans for the main western blotting are shown above.**

Supplementary tables

Supplementary Table 1. Characteristics of 142 patients with colorectal cancer included in the study.

Characteristics	No. of patients (N=142)
Gender (male/female)	80/62
Age, y (≦60/＞60)	60/82
Tumor size, cm (≦5/＞5)	54/88
Differentiation degree	
Low	18
Moderate	107
High	17
Stage	
Ⅰ	19
Ⅱ	55
Ⅲ	57
Ⅳ	11
Lymph node metastasis	
Postive	63
Negative	79

Supplementary Table 2. Mass spectrometry analysis of RHBDD1 interacting proteins.

No.	Accession	Description	Gene ID	Score	MW [kDa]
1	P01135	Protransforming growth factor alpha	7039	50.30	16.99
2	P68104	Elongation factor 1-alpha 1	1915	20.18	50.11
3	Q71U36	Tubulin alpha-1A chain	7846	10.21	50.10
4	O75340	Programmed cell death protein 6	10016	10.19	21.85
5	P49411	Elongation factor Tu, mitochondrial	7284	10.17	49.51
6	Q9UPN3	Microtubule-actin cross-linking factor 1	23499	10.17	62.00
7	O14744	Protein arginine N-methyltransferase 5	10419	10.16	72.64
8	O00483	NADH dehydrogenase 1 alpha subcomplex subunit 4	4697	10.15	9.36
9	Q9Y4H4	G-protein-signaling modulator 3	63940	10.14	17.85

Supplementary Table 3. Oligonucleotide sequences used for improved somatic cell knock-in strategy, gene knockdown strategy, reverse-transcription PCR and real-time PCR.

**Targeted knock-in of mutation in RHBDD1**			
**No.**	**Primer name**	**Forward (5' to 3')**	**Backward (5' to 3')**
1	Left arm	TGACCAGCTAGCAGATAATGTATGCTCGTGTC	CAACTAGTAAAATGACAGCAGCCACACC
2	Right arm	CAATCGATATGAAGTGTGGGTCCCTATC	TGACCAGTCGACAAAATTAGCTGGGCATGGTG
3	Screening	CTGGCAGTGTGGAAATTGG	AGGTGGGTGAATCACAAGGT
4	Cre	TTGCTGTTGCCGAATTTATG	CCTCCTCTTCAAGACGCAAA
5	Mutation identification	ATGCAACGGAGATCAAGAGG	TCCAGGGCAATAATGGTTGT
**Targeted knock-in of epitopes in RHBDD1**			
**No.**	**Primer name**	**Forward (5' to 3')**	**Backward (5' to 3')**
1	Left arm	CAACTAGTAGGAGAGAACAGAGTGGT	CAACTAGTCTGGCTATCGAATCTGTGAA
2	Right arm	TGACCAAGCTTGGTGGCATCTTGGGAAGACA	TGACCAAGCTTCGACTCAGACAGACAATCTG
3	Screening	GCAGGTGCTGTTGAGTCAAGT	TCATGGAACACAAGCACCAG
4	Cre	CACCCTACGGGTTTCATCTC	GAGCGATCTGGAGCAATACC
**Gene knockdown**			
**No.**	**Gene**	**siRNA 1#**	**siRNA 2#**
1	RHBDD1	GUAGAUGGUUUGCCUAUGUTT	GGAUUCUUGUUGGACUAAUTT
2	TACE	CAAAGAGACAGAGTGCTAGT	GAGAAGCTTGATTCTTTGC
3	TGFα	AACACUGUGAGUGGUGCCG	GAAGCAGGCCAUCACCGCCT
**Reverse-transcription PCR and real-time PCR**			
**No.**	**Gene**	**Forward (5' to 3')**	**Backward (5' to 3')**
1	*rhbdd1*	CTCTGGGACCGAGGAAATACC	ACCTCACTGGCTATCGAATCTGT
2	*β-catenin*	ACTGGCCTCTGATAAAGGCAACT	TAGTCGTGGAATAGCACCCTGTT

3	*β-actin*	AGGCCAACCGCGAGAAGAT	TCACCGGAGTCCATCACGAT
4	*gapdh*	TCAACGACCACTTTGTCAAGCTCA	GCTGGTGGTCCAGGGGTCTTACT
